# Seasonal and Diurnal Ammonia Emissions from Swine-Finishing Barn with Ground Channel Ventilation

**DOI:** 10.3390/ani15131892

**Published:** 2025-06-26

**Authors:** Jinho Shin, Heecheol Roh, Daehun Kim, Jisoo Wi, Seunghun Lee, Heekwon Ahn

**Affiliations:** 1 Department of Dairy Science, Chungnam National University, Daejeon 34134, Republic of Korea; sjh519@cnu.ac.kr (J.S.); eognsvv@naver.com (D.K.); 2 Department of Resource Innovation, Livestock Environmental Management Institute, Sejong 30127, Republic of Korea; nhc0902@gmail.com; 3 Animal Environment Division, National Institute of Animal Science, Rural Development of Administration, Wanju 55365, Republic of Korea; jisoowi@korea.kr; 4 Institute of Agricultural Science, Chungnam National University, Daejeon 34134, Republic of Korea; huny9261@cnu.ac.kr; 5 Division of Animal and Dairy Science, Chungnam National University, Daejeon 34134, Republic of Korea

**Keywords:** ammonia, swine barn, ground channel, seasonal variation, ventilation rate

## Abstract

This study evaluated the impact of a ground channel ventilation system on seasonal ammonia emissions in a swine-finishing barn over three distinct seasons: summer, late autumn, and winter. The ground channel system tempered inlet air, cooling it during summer and warming it during colder seasons, maintaining stable room temperatures despite external fluctuations. During summer, the ground channel reduced the incoming air temperature from 26.9 °C to 22.5 °C, contributing to steady barn temperatures (28.0 °C) and mitigating ammonia emissions, which reached 111.0 ± 23.6 g day^−1^ AU^−1^. In late autumn and winter, it warmed the inlet air from 4.7 °C and −0.7 °C to 8.1 °C and 6.8 °C, respectively, maintaining stable room temperatures (25.1 °C and 24.3 °C). Ammonia emissions remained consistent across seasons, with 125.0 ± 37.3 g day^−1^ AU^−1^ in late autumn and 107.1 ± 20.5 g day^−1^ AU^−1^ in winter. Thus, ammonia emissions showed no seasonal differences, highlighting the system’s effectiveness in balancing ventilation rates with emissions. During late autumn and winter, it improved air quality without compromising thermal comfort for the swine. In summer, the reduced ventilation demand lowered ammonia emissions, supporting the effective management of ammonia emissions year-round. Future research should investigate the system’s effects on other gases and slurry pit temperatures.

## Simple Summary

Swine barns often experience extreme temperature fluctuations, making it challenging to maintain optimal conditions for both animal welfare and reducing environmental impact. In hot weather, increased ventilation, while necessary for cooling, can inadvertently lead to higher ammonia emissions. Conversely, reducing ventilation in cold weather to conserve heat can worsen indoor air quality. This study investigated the effects of a ground channel ventilation system on ammonia emissions from swine barns. This system utilizes an underground air passage to moderate incoming air temperature. By pre-tempering the air, the system enables higher ventilation rates even in cold weather, improving air quality without compromising barn temperature. Furthermore, it reduces the need to maximize ventilation for cooling during hot weather, thereby helping to lower ammonia emissions. The results demonstrated that this system helped stabilize ammonia emissions across seasons. In conclusion, controlling inlet air temperature offers a promising strategy for managing ammonia emissions and promoting a more sustainable swine production system.

## 1. Introduction

If ammonia (NH_3_) emissions in swine barns are not properly managed, that could negatively impact swine productivity and workers’ health. Ammonia released into the air could cause soil acidification, groundwater contamination, water eutrophication, and global warming [1,2,3,4]. Moreover, ammonia reacts with HNO_3_, H_2_SO_4_, etc., as a precursor of particulate matter (PM_2.5_, PM_10_) [5,6,7]. In South Korea, approximately 262,000 tons of ammonia are generated annually, with about 76% coming from the agricultural and livestock sectors [8]. The livestock industry alone accounts for around 69% of the nation’s total ammonia emissions. Notably, the swine-farming sector contributes about 30% of the overall ammonia emissions in the country. A significant portion of this ammonia is released during the long-term manure storage in slurry pits. Therefore, it is essential to implement measures to reduce ammonia emissions in swine barns. To achieve this effectively, securing fundamental data on ammonia characteristics is necessary for proper control.

Ammonia emissions from swine barns are influenced by various factors, including barn structure, manure handling practice, feeding programs, and temperature fluctuations [9,10,11,12,13,14]. Changes in external air temperature significantly impact the ventilation rate within swine barns. Hinz and Linke [15] observed a swine-finishing barn equipped with a mechanical roof ventilation system, where the ventilation rate varied by approximately 5.8 times due to fluctuations in outside temperatures. The Midwest Plan Service (MWPS) suggested different ventilation rates for swine-finishing barns in cold and hot weather, with a maximum difference of up to approximately 187 m^3^ h^−1^ head^−1^ [16]. The American Society of Heating, Refrigerating, and Air-Conditioning Engineers (ASHRAE) also highlights a large seasonal ventilation gap for swine-finishing barns, with the recommended rate increasing from 18.0 m^3^ h^−1^ head^−1^ in winter to 216.0 m^3^ h^−1^ head^−1^ in summer—a difference of 198 m^3^ h^−1^ head^−1^ [17].

Numerous studies have investigated the relationship between ammonia emissions and ventilation rate variations in swine barns, considering seasonal and diurnal temperature fluctuations. These studies consistently demonstrate that ammonia emissions are significantly higher during summer and daytime, when temperatures are relatively high, compared to spring, winter, and nighttime. Heber et al. [18] reported ammonia emissions of 65 g day^−1^ AU^−1^ in spring and 147 g day^−1^ AU^−1^ in summer in a mechanically ventilated growing–finishing barn, where an animal unit (AU) is a standardized measure of livestock equivalent to approximately 500 kg of animal weight [19]. Dong et al. [20] observed similar trends in a naturally ventilated finishing barn, with emissions of 100 g day^−1^ AU^−1^ in spring and 148 g day^−1^ AU^−1^ in summer. Wi et al. [21] compared seasonal ammonia emissions in a conventional swine-finishing barn, reporting that emissions during summer (162 g day^−1^ AU^−1^) were approximately 1.9 times higher than those in autumn (86 g day^−1^ AU^−1^). Ma et al. [22] compared ammonia emissions in a swine-finishing barn during summer and winter across different manure removal cycles. Emissions were consistently higher in summer, with cumulative values of 116, 127, and 58 g N pig^−1^ compared to 86, 122, and 37 g N pig^−1^ in winter for the respective treatments. The authors attributed the increased summer emissions to higher room temperatures and ventilation rates.

However, in barns equipped with temperature control systems such as air-cooled condensers, mist–spray systems, cooling pads, and ground channel ventilation systems, the impact of ventilation rate on ammonia emissions is not significantly affected by seasonal and daily variations. Philippe et al. [23] measured ammonia emissions in a fattening barn that maintained optimal room temperature by preheating incoming air through an air-tempered hallway, reporting a 45 g day^−1^ AU^−1^ emission rate. This is only 45–65% of the emissions reported by Heber et al. [18] and Dong et al. [20]. Similarly, Stinn [24] measured ammonia emissions ranging from 32 to 60 g day^−1^ AU^−1^, which is 20–40% of the levels found by Heber et al. [18] and Dong et al. [20], in a swine barn equipped with cooling pads. However, when evaporative cooling is used to regulate indoor temperatures during the summer, managing humidity can be challenging without sufficient ventilation. Cao et al. [25] observed little seasonal difference in ammonia emissions in facilities using water pads in summer/ridge vents in winter, but high indoor humidity (82–95%) persisted, indicating humidity management challenges.

When the ground channel ventilation system is used in a swine barn for temperature control, hot outside air is precooled before entering the barn in summer, and cold outside air is preheated before entering in winter [26,27]. There has been a growing interest in sustainable livestock farming in recent years, with a particular focus on reducing environmental impacts such as ammonia emissions. Ground channel ventilation systems have been recognized for their potential to improve energy efficiency and humidity control in swine barns. While the economic advantages of these systems have been well-documented, research on their effectiveness in mitigating ammonia emissions remains limited [28,29,30]. This study aims to address this gap by assessing ammonia emissions in a windowless swine-finishing barn equipped with a ground channel ventilation system. By monitoring real-time data on ventilation rates and ammonia concentrations, we evaluated how variations in external temperature affect ammonia emissions. This approach provides valuable insights into the environmental performance of ground channel ventilation systems, contributing to more sustainable swine farming.

## 2. Materials and Methods

### 2.1. Swine Barn Design and Ventilation System Description

**Figure 1:**
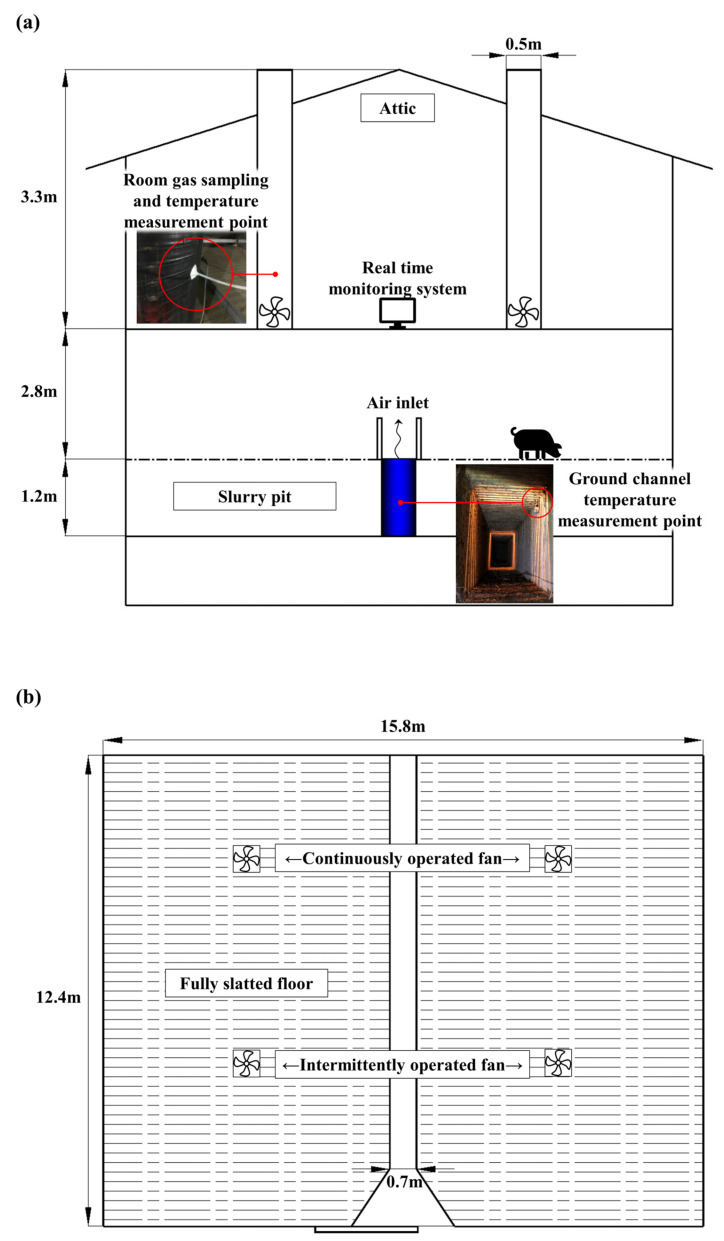
Section view (**a**) and plan view (**b**) of the swine-finishing barn used in this study.

**Figure 2:**
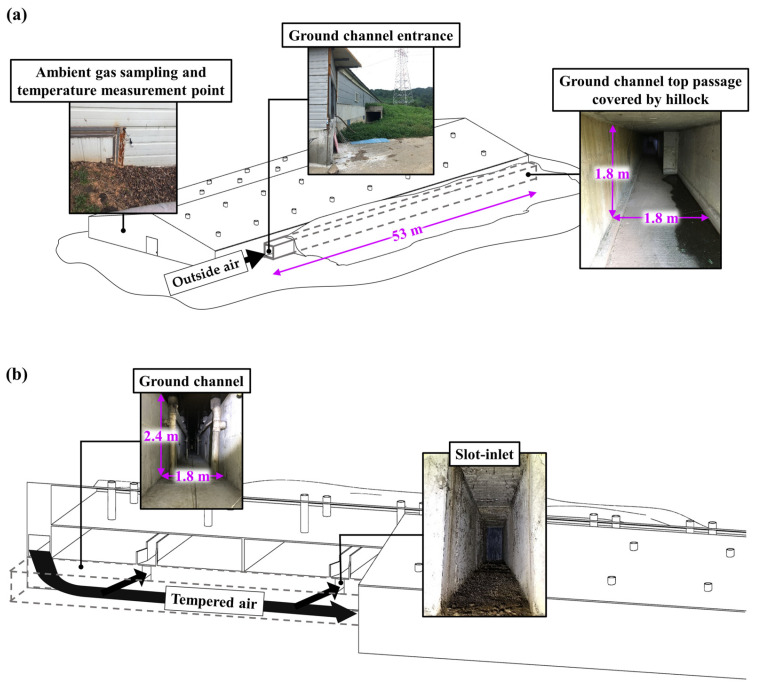
The ground channel ventilation system of the swine-finishing barn used for this study: (**a**) Top passage of the ground channel covered by hillock; (**b**) Underground part of the ground channel connected to each finishing room.

This study was conducted in a windowless swine-finishing barn consisting of eight rooms and a ground channel ventilation system (Figure 1). Each room was fully enclosed and independently managed, with identical ventilation systems, internal structures, and construction materials. Each room had a pig-rearing area of approximately 187.2 m^2^ and was divided into two pens of equal size. The ground channel system consists of a top passage and a bottom section, with a total volume of 452.5 m^3^ (Figure 2). The top passage accounts for 171.7 m^3^ (height: 1.8 m, width: 1.8 m, length: 53 m), while the bottom section beneath the barn contributes 280.8 m^3^ (height: 2.4 m, width: 1.8 m, length: 65 m). This experiment was conducted in one of the eight rooms, which remained the same across all seasons. This room was selected based on comparable animal and housing conditions throughout the study period to ensure consistent seasonal comparisons. The portion of the ground channel associated with this room had a total volume of 374.8 m^3^. Depending on the season, the outside air was either precooled or preheated before entering the finishing rooms through a slot inlet located on the floor.

The experiments in late autumn and winter were conducted for 16 days each in 2019, from November 17 to December 2 (late autumn, average outside temperature: 4.7 ± 1.6 °C) and from January 12 to January 27 (winter, average outside temperature: −0.7 ± 2.1 °C). The summer experiment was carried out over 14 days in 2020, from August 20 to September 2 (summer, average outside temperature: 26.9 ± 1.0 °C). Daytime and nighttime were classified as 07:00–19:00 and 19:00–07:00, respectively. During the experiment period, finishing pigs were used, with body weights ranging from 74 to 86 kg in summer (171 pigs), 74 to 87 kg in late autumn (161 pigs), and 66 to 79 kg in winter (176 pigs). They were fed ad libitum with finishing-phase diets in which the crude protein content was maintained at 17.0% on a dry basis, while crude fiber and fat contents were kept below 5.0% and 3.0%, respectively. The stocking density was maintained within the recommended range of 0.8–1.3 m^2^ head^−1^, as specified by regulatory standards for finishing pigs in the Republic of Korea [31,32].

Each room was equipped with four ventilation fans, each with a diameter of 500 mm. Two fans continuously operated with variable speed, automatically adjusting to maintain the set room temperature of 25 °C. The remaining two fans were intermittently operated fans, which were run at full capacity during the sanitization period, lasting about a week after the pigs were sent to market. Only two continuously operated fans were used during the experimental periods (Figure 1). Manure excreted during each breeding cycle accumulated in a 1.2 m deep slurry pit (226 m^3^) located beneath each room, which featured a fully slatted floor (188 m^2^). At the end of each production cycle, the slurry pit was fully emptied before the next batch of pigs was introduced. In the summer, late autumn, and winter experiments, monitoring began 16, 15, and 6 days after pig placement, respectively. As a result, at the start of each measurement period, manure accumulation in the slurry pit was approximately 7%, 6%, and 3% of its total volume.

### 2.2. Ammonia, Ventilation Rate, and Temperature

A real-time monitoring system (OMS-200, Smart Control & Sensing Inc., Daejeon, Rep. of Korea) was used to continuously monitor ammonia concentration, ventilation rate, and temperature within the finishing room. Before installation of the real-time monitoring system in the farm, an electrochemical gas sensor (NH_3_/CR-200, Membrapor Co., Wallisellen, Switzerland) was calibrated using ammonia standard gases (ammonia 0, 6, 12, 24, and 48 mL m^−3^). The detailed specifications of the ammonia sensor are presented in Table S1. Ammonia concentration in the finishing room was measured by collecting air samples from the duct of a continuously operating exhaust fan at hourly intervals. Ambient ammonia concentration was also measured hourly. Before each measurement, the gas sensor in the monitoring system was flushed for ten minutes to remove any residue gas from previous measurements, ensuring the accuracy of the following readings. The measurement locations for ammonia concentration are shown in Figure 1 and Figure 2.

Ventilation rates were measured using a modified SWEAP system, as described by DeVoe et al. [33]. The modified SWEAP system employed five hot-wire anemometers (Wind Sensor Rev. P, Modern Device, Providence, RI, USA) mounted at regular intervals across the 500 mm diameter fan, ensuring that each sensor measured an equal portion of the cross-sectional area. The system traversed this cross-section at 127 ± 13 mm s^−1^, as described by DeVoe et al. [33], to measure fan air velocity. The output signals of the hot-wire anemometers were measured using an NI 9205 module (National Instruments, Austin, TX, USA). Each measurement collected 110 air velocity data points per session, and a representative fan airflow profile is presented in Figure S1. Subsequently, the airflow rate of the ventilation fan was determined by multiplying the air velocities measured at each measurement point by the corresponding cross-sectional areas. Ventilation rate measurement was conducted under standard pig housing conditions. During this process, the differential static pressure was also measured using a differential pressure sensor (GMH 3161-002, GHM Messtechnik GmbH, Regenstauf, Germany). The measured ventilation rates corresponding to the differential static pressure in the pig room are presented in Figure S2. The ventilation fan-operating rate was determined through real-time monitoring of fan-operating voltage, which was pre-measured at various operating rates.

Temperatures in the finishing room, ground channel, and ambient air were measured hourly using temperature sensors (MHTP-485S, Econarae, Yongin-si, Rep. of Korea). The detailed specifications of the temperature sensor are presented in Table S2. The temperature measurement points at each location are shown in Figure 1 and Figure 2.

Ammonia emissions were expressed per animal unit, where one AU represents 500 kg of live body weight. Daily ammonia emissions were calculated as the sum of the hourly emissions, determined by the following equation [34]:

$$\begin{array}{l} Ammonia\;emission\left(g\;{hr}^{-1}{AU}^{-1}\right)\\ \;\;\;\;\;\;=(C_{E}\times V\times \frac{273.15\times MW}{\left(273.15+T_{E}\right)\times 22.4\times 10^{3}}\\ \;\;\;\;\;\;-C_{A}\times V\times \frac{273.15\times MW}{\left(273.15+T_{A}\right)\times 22.4\times 10^{3}})\times 60\\ \;\;\;\;\;\;÷\;number\;of\;animal\;unit \end{array}$$

where

CE = Ammonia concentration of exhausted air (mL m^−3^);
V = Room ventilation rate (m^3^ min^−1^);
MW = Molecular weight of ammonia gas (g mol^−1^);
TE = Exhaust air temperature (°C);
CA = Ammonia concentration of ambient air (mL m^−3^);
TA = Ambient air temperature (°C);
Number of animal units = Number of pigs × Average body weight (kg) ÷ 500 kg.

### 2.3. Statistical Analysis

Seasonal differences in data were analyzed using a one-way analysis of variance (ANOVA) with Origin Pro software (OriginLab, version 8.1), which is appropriate for comparing multiple groups. Diurnal differences in each season were analyzed using two-sample *t*-tests with Origin Pro software (OriginLab, version 8.1), as this method is suitable for comparing two independent groups (day vs. night). Before conducting ANOVA and *t*-tests, normality was assessed, and homogeneity of variance was verified where applicable. A *p*-value of less than 0.05 was considered statistically significant.

## 3. Results and Discussion

### 3.1. Seasonal Characteristics of Temperature and Ventilation Rate

**Figure 3:**
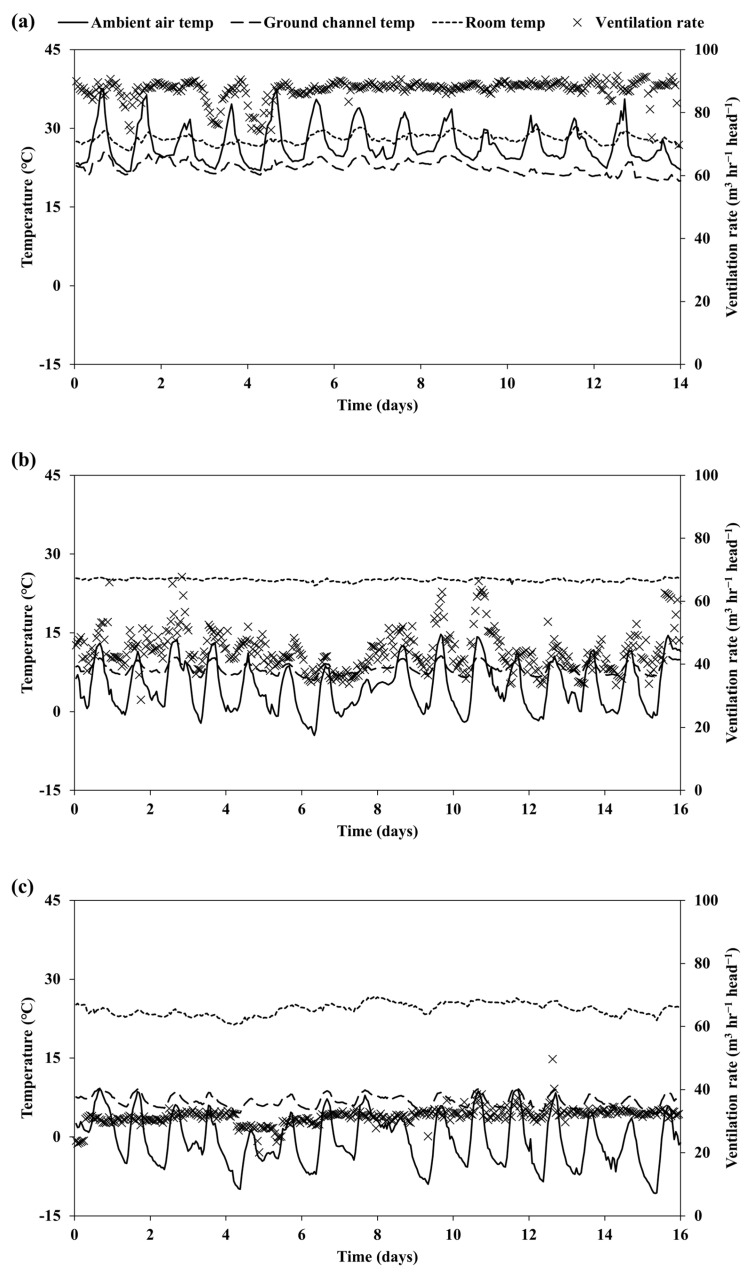
Ventilation rate, ambient, ground channel, and room temperature in summer (**a**), late autumn (**b**), and winter (**c**).

Figure 3 shows the seasonal variations in ambient air, ground channel, room temperatures, and ventilation rate. During the summer, the ambient air temperature fluctuated significantly throughout the day, peaking at midday. The ground channel temperature remained lower than the ambient air, providing a cooling effect that helped stabilize the room temperature, which stayed relatively constant. The ventilation rate was highest in summer to manage the heat. As autumn progressed, the ambient air temperature became cooler. The ground channel temperature remained higher than the ambient air, helping to moderate the cooler conditions. This allowed for the room temperature to stay stable and slightly cooler than in summer. The ventilation rate was reduced compared to summer. In winter, the ambient air temperature was the lowest. The ground channel acted as a strong buffer, maintaining a much higher temperature than the ambient air, which helped keep the room temperature stable and warm. The ventilation rate was lowest during this season to conserve heat while allowing for minimal air exchange to maintain indoor air quality.

The difference between the average ambient and ground channel temperatures varied across seasons (Table 1). In summer, the ground channel temperature (22.5 °C) was lower than the ambient temperature (26.9 °C). In contrast, in late autumn and winter, the ground channel temperatures (8.1 °C and 6.8 °C, respectively) were notably higher than the ambient temperatures (4.7 °C and −0.7 °C, respectively). This indicated that the ground channel system acted as a thermal buffer, reducing the temperature fluctuations entering the room where the pigs were housed. As a result, despite the substantial changes in ambient temperature across seasons, the room temperature remained relatively stable: 28.0 °C in summer, 25.1 °C in late autumn, and 24.3 °C in winter.

Without such a buffering system like the ground channel, if cold ambient air had been directly introduced into the room during winter, it would have resulted in significant internal energy loss, requiring more energy for room temperature maintenance. To avoid excessive heat loss, minimum ventilation is typically recommended during winter. This aligns with findings from Xie et al. [35], which demonstrated that in cold outdoor conditions, heating systems are needed to prevent indoor temperature drops. Additionally, ventilation must be maintained at a minimum level to introduce fresh air while avoiding excessive heat loss. However, this often led to poor air quality inside the pig rooms, as reduced ventilation could result in the accumulation of harmful gases. In this experiment, however, the swine facility with a ground channel system allowed for higher winter ventilation rates than conventional swine facilities. MWPS and ASHRAE recommend a minimum winter ventilation rate of 17–18 m^3^ h^−1^ head^−1^ for conventional swine barns (Table 1). Qi et al. [36], highlighting the need for energy-efficient ventilation design in winter pig housing, reported an even lower average of 14.4 m^3^ h^−1^ head^−1^. In contrast, the facility in this study—equipped with a ground channel system—achieved a significantly higher rate of 31.5 ± 1.7 m^3^ h^−1^ head^−1^, nearly double the recommended values.

Despite this higher ventilation rate, the ground channel system effectively buffered the air temperature, maintaining the room temperature without compromising the thermal comfort of the pigs. This higher ventilation rate during winter proved beneficial for maintaining better air quality inside the pig rooms, highlighting the advantages of the ground channel system in improving both temperature control and air quality in swine barns compared to conventional facilities.

According to the Korea Meteorological Administration [37], the 10-year (2014–2023) average ambient temperatures for Korea are 24 °C in summer, 15 °C in autumn, and 1 °C in winter. The recorded average ambient temperatures at the swine facility used in this study were 27 °C in summer, 4.7 °C in late autumn, and −0.7 °C in winter. The significantly lower temperature in autumn compared to the national average can be attributed to the study being conducted primarily during late autumn. Table 2 compares the ventilation rates reported in traditional windowless swine barns without temperature buffering systems to the results from this study, which used a temperature buffering system. The comparison is categorized by external air temperature: summer (above 15 °C), autumn (4–15 °C), and winter (below 4 °C). The results indicate that ventilation rates were significantly higher in summer and autumn than in winter, and the temperature buffering system contributed to maintaining more stable ventilation.

In previous studies, ventilation rates in traditional systems were reported to be 4.4–12 times higher in summer compared to winter and 2.7–3.6 times greater in autumn compared to winter (Table 2). In contrast, the ventilation rate in this study, using a temperature buffering system, was only 2.8 times higher in summer and 1.4 times higher in autumn compared to winter. These results indicate that the temperature buffering system helped maintain more stable ventilation rates, with a much smaller increase in ventilation compared to traditional systems. This suggests that the temperature buffering system used in this study provided better control over internal environmental conditions, reducing the need for dramatic increases in ventilation rates as seen in traditional systems.

Research on swine barns equipped with inlet air temperature control systems has also shown smaller seasonal variations in ventilation rates compared to conventional barns. According to Aarnink et al. [9], in a swine barn with underground heat exchange tubes, the ventilation rate in the rearing room during mild weather (spring) was 1.3 times that of winter. In the same study, ventilation rates in the rearing and fattening rooms during hot weather (summer) were 2.3–2.7 times greater than in cold weather (winter). Similarly, Costa and Guarino [38] reported that in the farrowing, where an underground tunnel and attic conditioned outside air before entering, the ventilation rate during hot weather (outside temperature: 23 °C) was 2.5 times higher than during cold weather (outside temperature: 3 °C). Additionally, in a swine barn where outside air was tempered in a service corridor before flowing into the fattening pen, the summer ventilation rate was 2.7 times higher than the winter rate [11].

**Table 2:** Comparison of seasonal ventilation rate changes in conventional swine barns from previous studies and this study’s results. (Mean ± S.D.).

	Growth Stage	Stocking Density (m^2^ head^−1^)	Ambient Temperature (°C)	Ventilation Rate (m^3^ h^−1^ head^−1^)	Ventilation Rate	
					Autumn (Compared to Winter)	Summer (Compared to Winter)
This study	Finisher	1.09	26.9 ± 1.0	87.1 ± 1.9	1.4 times	2.8 times
		1.16	4.7 ± 4.5	44.3 ± 3.8		
		1.06	−0.7 ± 4.8	31.5 ± 1.7		
MWPS [16]	Finisher	0.83	Hot weather	203.9	3.4 times	12.0 times
		0.74	Mild weather	59.5		
			Cold weather	17.0		
ASHRAE [17]	Finisher	0.74	Hot weather	216.0	-	12.0 times
			Cold weather	18.0		
Sun et al. [39] *	Fattening	1.02	19.4 ± 6.4	161.0	-	4.4–7.2 times
		1.04	−1.6 ± 3.4	36.0		
		0.97	−9.7 ± 3.6	22.4		
Blunden et al. [40] *	Finisher	0.83	22.3 ± 4.9	68.6	3 times	-
		0.87	5.5 ± 4.8	23.1		
Costa and Guarino [38] *	Gestation	2.02	24.39	116.2	2.7 times	6.0 times
		2.29	8	43.6		
		2.02	3.13	19.4		
	Fattening	0.86	24.4	81.4	3.6 times	-
		0.86	7.1	22.9		
Rumsey et al. [41] *	Fattening	Not available	26.0 ± 4.1	119.6	3.6 times	-
			11.3 ± 6.2	33.0		

Note: Previous studies marked with an asterisk (*) are derived from swine barns using mechanical ventilation without inlet air control. Detailed housing characteristics and manure handling practices for these studies are provided in Table S6 of Supplementary Material.

### 3.2. Seasonal Ammonia Concentration and Emission

Table 3 shows the seasonal variations in ventilation rate, ammonia concentration, and emission. Ventilation rates varied significantly across seasons, with the highest rate observed in summer, followed by late autumn, and then winter (*p* < 0.05). This increased ventilation in summer led to a dilution effect, resulting in a lower ammonia concentration (11.0 ± 2.1 mL m^−3^), which was approximately 45% of the concentration observed in late autumn (24.2 ± 6.0 mL m^−3^) and about 41% of that in winter (26.8 ± 4.5 mL m^−3^), both showing statistically significant differences (*p* < 0.05). In contrast, the lower ventilation rates in late autumn and winter resulted in higher ammonia concentrations of 24.2 ± 6.0 mL m^−3^ and 26.8 ± 4.5 mL m^−3^, respectively.

Previous studies have generally shown that ammonia emissions from swine barns without inlet air control systems tend to be higher during warmer weather compared to colder periods [9,18,42,43]. Heber et al. [18] found that in a grow–finish swine barn with manure stored in a 2.4 m deep pit and ventilation provided through the sidewall and pit fans, ammonia emissions during warm weather (ambient temperature: 20.0–21.9 °C) were approximately 1.9 to 2.2 times higher than during mild weather (ambient temperature: 8.4–12.0 °C). Similarly, Sun et al. [39] observed that ammonia emissions in a finishing barn with a fully slatted floor and sidewall ventilation fans were 1.5 to 1.8 times higher during warm weather (19.4 °C) compared to cold weather (−1.6 °C to −9.7 °C). Harper et al. [42] also reported that ammonia emissions in a finishing barn with gutters inside the pit that were flushed using recycled lagoon water were 3.2 times higher in summer than in winter.

In the present study, however, ammonia emissions remained relatively constant across seasons, ranging from 107 to 125 g day^−1^ AU^−1^ (*p* > 0.05). This can be attributed to using a ground channel system, which moderated the temperature of the incoming air.

In conventional swine barns, ventilation is often kept minimal to retain heat during winter. With reduced ventilation, ammonia in the barn is not effectively removed, resulting in much higher indoor ammonia concentrations. In contrast, the ground channel system allowed for the ventilation rates to be 1.8–1.9 times higher than conventional practices [16,17]. As a result, the ground channel-ventilated barn showed a markedly more moderate seasonal increase in ammonia levels (Table S3). The winter ammonia concentration was only about 2.4 times higher than summer, compared to the 2.6 to 20.9 times increase typically observed in conventional barns. This demonstrates that the ground channel system can alleviate the deterioration in indoor air quality commonly caused by maintaining minimal ventilation during winter, even under frigid outside conditions.

In this study, the increase in summer ventilation rates was relatively modest compared to conventional swine barns, as discussed in Section 3.1, due to the precooling of incoming air through the ground channel system. Although direct comparisons are limited by differences in slurry pit manure and sedimentation, ventilation strategies, and management practices, ammonia emissions in summer were approximately 0.69–0.91 of the levels reported in previous studies (Table S4). However, emissions were 2.08–2.95 times higher compared to certain other studies, which could be partially attributed to the relatively high crude protein (CP) content in the feed used in this study. The CP content in the feed in this study was 17.0% (dry basis), which is approximately 3.2 percentage points higher than the average CP content of 13.8% recommended by the National Research Council [44], calculated by multiplying the total nitrogen requirement for 50–75 kg pigs by the conventional CP conversion factor of 6.25. Niyazov and Ostrenko [45] reported that reducing CP levels in pig feed from 172 g kg^−1^ to 151 g kg^−1^ (growing stage) and 142 g/kg (finishing stage) reduced nitrogen excretion by 24.4–33.8% in urine and 20.7–36.0% in feces. According to the meta-analysis conducted by Sajeev et al. [46], a one-percentage-point reduction in CP content in pig diets resulted in an approximately 11 ± 6% decrease in ammonia emissions. Therefore, the high CP content in the feed may have increased nitrogen levels in the manure, increasing the amount of nitrogen available for ammonia formation.

In conclusion, the ground channel ventilation system moderated incoming air temperature, providing more stable ammonia emissions throughout the year than conventional swine barns. This consistency makes it easier to manage ammonia emissions. In addition, to assess the potential impact of increasing pig body weight and manure accumulation in the slurry pit over time, the monitoring periods were divided into two halves (early and late phases). Ammonia emissions were then evaluated for each phase. However, no significant increase in emissions was observed during the latter periods (Table S5). While greater manure accumulation is expected to elevate ammonia volatilization, such effects were not evident within the relatively short study durations. It is expected that emissions would increase over more extended manure storage periods.

### 3.3. Variation in Diurnal Ammonia Concentration and Emission

The effect of a ground channel on diurnal barn temperature and ventilation rate for each season is illustrated in Table 4. The differences between the day and night ambient temperatures were 5.2 ± 1.7 °C, 4.6 ± 1.1 °C, and 4.4 ± 1.3 °C in summer, late autumn, and winter, respectively. In contrast, for the ground channel, the temperature differences were minimal, at 0.7 ± 0.5 °C in summer and 1.1 ± 0.3 °C in late autumn and winter. These minimal diurnal temperature variations indicate the ground channel’s buffering effect, reducing the need for dynamic changes in ventilation rates. Demmers et al. [47] reported that the ventilation rate showed diurnal patterns, with a high ventilation rate during the day and a low ventilation rate at night to manage the fluctuating barn temperatures. However, despite ambient temperature differences of 4.4–5.2 °C between day and night in all seasons, the ventilation rates in this study did not show significant diurnal variations (*p* > 0.05), likely due to the stabilizing effect of the ground channel ventilation system.

**Figure 4:**
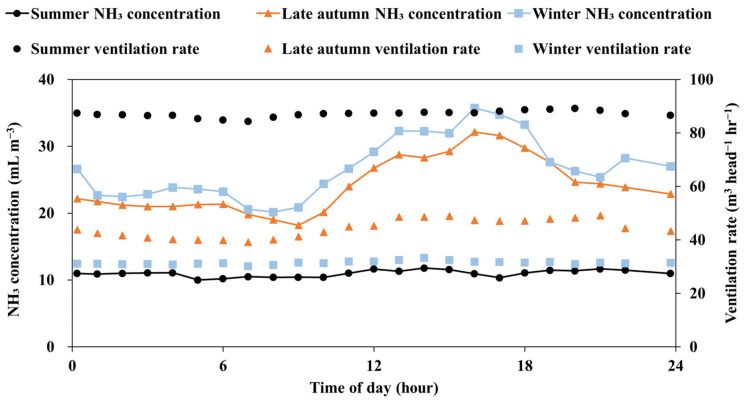
Diurnal ammonia concentration and ventilation rate profile of each season based on averaged hourly data over the entire measurement period.

Ammonia concentration generally decreases during the day and increases at night due to changes in ventilation rates [39,48]. However, in this experiment, the diurnal ammonia concentration pattern differed. Figure 4 presents the average hourly values of ammonia concentration and ventilation rate, calculated by averaging the corresponding hour across all days within each seasonal monitoring period. Table 5 shows the seasonal averages of ventilation rate, ammonia concentration, and ammonia emission rate during daytime (07:00–19:00) and nighttime (19:00–07:00), based on daily means calculated for each period. As shown in Figure 4 and Table 5, ammonia concentration remained stable throughout the day in summer, whereas daytime concentrations were higher than nighttime concentrations in late autumn and winter, with statistically significant differences observed only in winter (*p* < 0.05). Higher daytime ammonia concentrations in late autumn and winter likely resulted from daytime pig activities, such as eliminative and feeding behaviors [49,50,51]. Blanes-Vidal et al. [49] reported that diurnal variations in ammonia emissions were strongly correlated with fluctuations in pig activity (R^2^ = 0.94), underscoring the significant impact of pig behavior on ammonia emissions in swine barns. Groenestein et al. [50] confirmed that feeding activity in swine barns significantly contributes to ammonia emission fluctuations influenced by indoor temperature and animal activity. Guo et al. [51] reported that pig eliminative behavior peaks between 13:00 and 14:00, following a distinct diurnal pattern with increased activity during the day. These pig activities become more noticeable when ventilation rates decrease. In contrast, during periods of high summer ventilation, the substantial effect of ventilation rate on barn ammonia concentration overshadowed the influence of daytime pig activities.

Regarding ammonia emissions, Table 5 shows that in summer, ammonia emissions remained constant throughout the day due to stable ventilation rates and ammonia concentrations. In contrast, in late autumn and winter, emissions were also higher during the day than at night, reflecting the diurnal variations in ammonia concentration. Specifically, emissions were 5.6 ± 1.8 g h^−1^ AU^−1^ during the day and 4.8 ± 1.4 g h^−1^ AU^−1^ at night in late autumn, while in winter, emissions were 4.8 ± 1.1 g h^−1^ AU^−1^ during the day and 4.1 ± 0.7 g h^−1^ AU^−1^ at night. Notably, statistically significant differences in ammonia emissions between day and night were only observed during winter (*p* < 0.05), when diurnal variations in ammonia concentration were also observed. This aligns with Guo et al. [52], who stated that when ventilation rate differences are minimal, variations in ammonia emissions are primarily determined by ammonia concentration.

## 4. Conclusions

This study demonstrated that a ground channel system mitigated the effects of seasonal ambient temperature fluctuations, reducing ammonia emission variability seen in conventional barns. By tempering the inlet air temperatures year-round, the system cooled the air in summer and warmed it in late autumn and winter, stabilizing internal barn conditions and minimizing energy consumption for heating and cooling. The ground channel system’s cooling effect reduced ammonia emissions in summer, while increased ventilation in winter (buffered by the ground channel) caused only a slight rise in emissions. Consequently, ammonia emissions remained more consistent throughout the year. These findings highlight the ground channel system’s benefits for emission management and energy efficiency. Its effectiveness is expected to be maximized in climates with wide thermal variation, where temperature-buffering systems can have the greatest impact on maintaining stable barn environments. Future research should explore its impact on other odor compounds and greenhouse gases and how tempered air affects slurry temperature and gas volatilization.

## Figures and Tables

**Table 1:** Effect of the ground channel ventilation system on inlet air temperature. (Mean ± S.D.).

	Summer (*n* = 14)	Late Autumn (*n* = 16)	Winter (*n* = 16)
Ambient temperature (°C)	26.9 ± 1.0 ^a^	4.7 ± 1.6 ^b^	−0.7 ± 2.1 ^c^
Ground channel temperature (°C)	22.5 ± 1.0 ^a^	8.1 ± 0.4 ^b^	6.8 ± 0.5 ^c^
Room temperature (°C)	28.0 ± 0.6 ^a^	25.1 ± 0.2 ^b^	24.3 ± 1.0 ^c^
Ventilation rate (m^3^ h^−1^ head^−1^)	87.1 ± 1.9 ^a^	44.3 ± 3.8 ^b^	31.5 ± 1.7 ^c^

^a–c^ Different superscripts in the same row mean that each group is significantly different (*p* < 0.05).

**Table 3:** Comparison of ventilation rate, ammonia concentration, and ammonia emission between summer, late autumn, and winter. (Mean ± S.D.).

	Summer (*n* = 14)	Late autumn (*n* = 16)	Winter (*n* = 16)
Ventilation rate (m^3^ h^−1^ head^−1^)	87.1 ± 1.9 ^a^	44.3 ± 3.8 ^b^	31.5 ± 1.7 ^c^
NH_3_ concentration (mL m^−3^)	11.0 ± 2.1 ^a^	24.2 ± 6.0 ^b^	26.8 ± 4.5 ^b^
NH_3_ emission (g day^−1^ AU *^−1^)	111.0 ± 23.6 ^a^	125.0 ± 37.3 ^a^	107.1 ± 20.5 ^a^

^a–c^ Different superscripts in the same row mean that each group is significantly different (*p* < 0.05). * AU: 500 kg live body weight.

**Table 4:** Diurnal variation in temperature and ventilation rate during each season. (Mean ± S.D.).

	Summer (*n* = 14)		Late Autumn (*n* = 16)		Winter (*n* = 16)	
	Day	Night	Day	Night	Day	Night
Ambient temperature (°C)	29.4 ± 1.7 ^a^	24.3 ± 0.8 ^b^	7.0 ± 1.7 ^a^	2.4 ± 1.8 ^b^	1.6 ± 2.0 ^a^	−2.9 ± 2.3 ^b^
Ground channel temperature (°C)	22.8 ± 1.2 ^a^	22.2 ± 0.8 ^a^	8.7 ± 0.4 ^a^	7.6 ± 0.4 ^b^	7.4 ± 0.5 ^a^	6.3 ± 0.6 ^b^
Room temperature (°C)	28.4 ± 0.7 ^a^	27.7 ± 0.6 ^b^	25.1 ± 0.2 ^a^	25.0 ± 0.2 ^a^	24.4 ± 1.0 ^a^	24.2 ± 1.1 ^a^
Ventilation rate (m^3^ h^−1^ head^−1^)	87.2 ± 1.4 ^a^	87.1 ± 2.6 ^a^	45.1 ± 4.9 ^a^	43.5 ± 3.3 ^a^	31.8 ± 1.9 ^a^	31.2 ± 1.7 ^a^

^a,b^ Different superscripts in the same row mean that each group is significantly different (*p* < 0.05).

**Table 5:** Comparison of ventilation rate, ammonia concentration, and emission between summer, late autumn, and winter. (Mean ± S.D.).

	Summer (*n* = 14)		Late autumn (*n* = 16)		Winter (*n* = 16)	
	Day	Night	Day	Night	Day	Night
Ventilation rate (m^3^ h^−1^ head^−1^)	87.2 ± 1.4 ^a^	87.1 ± 2.6 ^a^	45.1 ± 4.9 ^a^	43.5 ± 3.3 ^a^	31.8 ± 1.9 ^a^	31.2 ± 1.7 ^a^
NH_3_ concentration (mL m^−3^)	11.0 ± 2.1 ^a^	11.0 ± 2.3 ^a^	25.6 ± 6.7 ^a^	22.8 ± 5.6 ^a^	28.5 ± 5.3 ^a^	25.0 ± 4.1 ^b^
NH_3_ emission (g h^−1^ AU *^−1^)	4.5 ± 1.0 ^a^	4.7 ± 1.0 ^a^	5.6 ± 1.8 ^a^	4.8 ± 1.4 ^a^	4.8 ± 1.1 ^a^	4.1 ± 0.7 ^b^

^a,b^ Different superscripts in the same row mean that each group is significantly different (*p* < 0.05). * AU: 500 kg live body weight.

## Data Availability

The original contributions presented in this study are included in this article. Further inquiries can be directed to the corresponding author.

## Supplementary Materials

The following supporting information can be downloaded at: https://www.mdpi.com/article/10.3390/ani15131892/s1, Table S1. Ammonia sensor specification. Table S2. Temperature sensor specification. Table S3. Comparison of winter-to-summer ammonia concentration ratios in swine barns [39,53,54,55]. Table S4. Comparison of ammonia emission rates during summer [11,18,21,39,55,56]. Table S5. Seasonal ammonia emissions and manure accumulation in the slurry pit during different monitoring periods. Table S6. Housing characteristics and manure handling practices in previous studies referenced in Table 2 [38,39,40,41]. Figure S1. Example of fan airflow profile. (a) Air velocity distribution (b) Cumulative and strip total airflow rates. Figure S2. Relationship between differential static pressure and ventilation rate.
