# Supplementary material for: Seasonal and Diurnal Ammonia Emissions from Swine-Finishing Barn with Ground Channel Ventilation
Source: Animals (Basel). 2025 Jun 26;15(13):1892. doi: 10.3390/ani15131892 (PMC12248801; doi:10.3390/ani15131892)
Supplement: Supplementary file 1 [file animals-15-01892-s001.zip › animals-3657110-supplementary.pdf]

Supplementary materials

Seasonal and diurnal ammonia emission from swine finishing barn with ground channel ventilation

Table S1. Ammonia sensor specification.

**Operation principle**	**3-Electrode Electrochemical**
**Nominal range**	0–100 ppm
**Output signal**	112 ± 40 nA/ppm
**Resolution**	< 0.5 ppm
**T90 Response time**	< 35 s
**Repeatability**	< 3% of signal

Table S2. Temperature sensor specification.

**Operation principle**	**Intergral PTAT silicon transistor**
**Scale**	-20°C to 80°C
**Accuracy**	± 0.4°C from -10°C to 70°C
**Resolution**	0.1°C

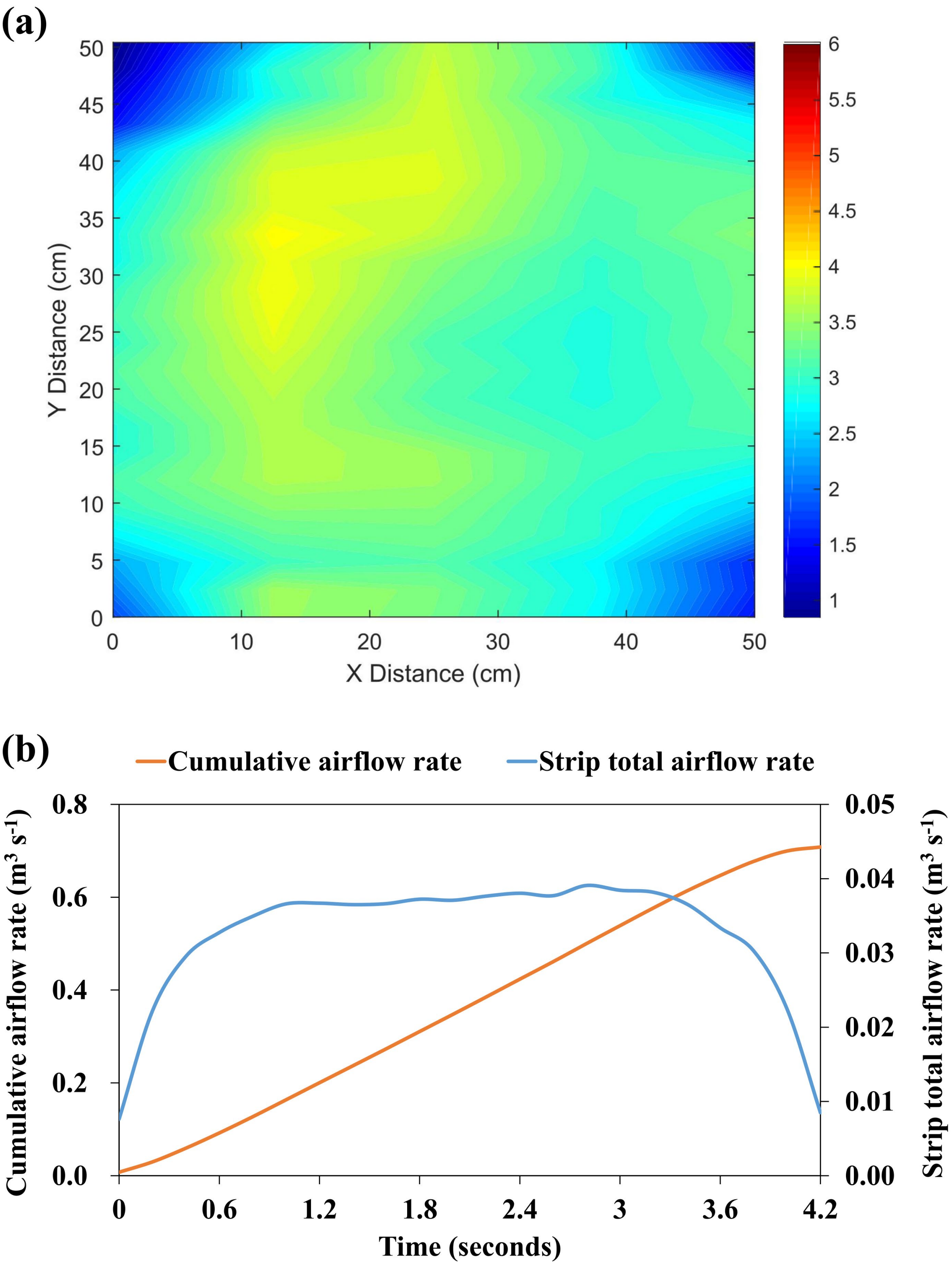

Figure S1. Example of fan airflow profile. (a) Air velocity distribution (b) Cumulative and strip total airflow rates.

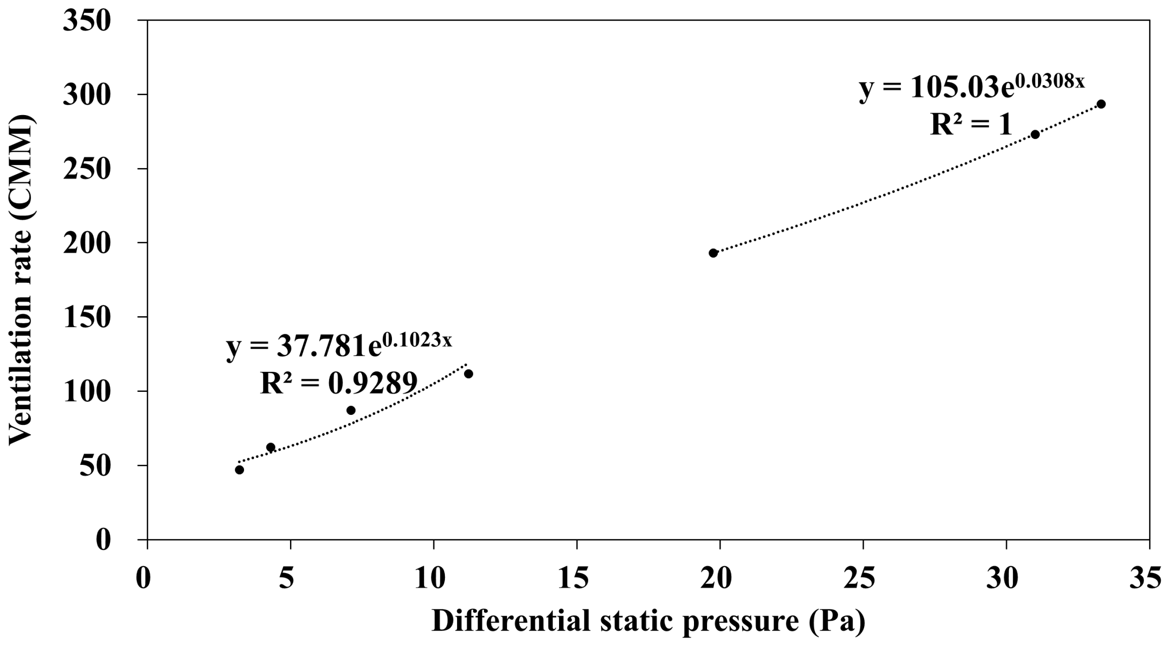

Figure S2. Relationship between differential static pressure and ventilation rate.

Table S3. Comparison of winter-to-summer ammonia concentration ratios in swine barns.

	Growth stage	Stocking density (m^2 $head^{-1}$)	Season (ambient temperature (°C))	Floor type	Ventilation type	Ammonia concentration (ppm)	Winter-to-summer ammonia ratio
This study	Finishing pig	1.09	Summer (26.9 ± 1.0)	Fully slatted floor	Mechanical ventilation	11.0	2.4
		1.06	Winter (-0.7 ± 4.8)			26.8	
Choi, *et al.* [1]	Fattening pig	N/A†	Summer (N/A)			0.9	20.9
			Winter (N/A)			19.2	
Jerez, *et al.* [2]	Wean-to-finish pig	N/A	Summer (16.5–22.1)			1.2	8.0
			Winter (-9.7–13.2)			9.6	
Sun, *et al.* [3]	Fattening pig	0.91	Summer (19.4 ± 6.4)			9.0	3.6
		0.87	Winter (-9.7 ± 3.6)			32.0	
Van Ransbeeck, *et al.* [4]	Fattening pig	N/A	Summer–Autumn (11.3–20.3)			8.9–11.4	2.6‡
			Autumn–Winter (0.1–3.2)			2.5–5.9	

† N/A = Not available.

‡ This ratio represents the mean of three experimental phases reported in the referenced study.

Table S4. Comparison of ammonia emission rates during summer.

	Growth stage	Stocking density (m^2 $head^{-1}$)	Temperature (℃)		Manure handling practice	Ammonia emission rate
			Ambient	Room		
This study	Finishing pig	1.09	26.9	28.0	Slurry pit (depth: 1.2 m), Fully slatted	111.0 g day^{-1} AU^{-1}
						17.7 g day^{-1} $head^{-1}$
Sun, et al. [3]	Fattening pig	0.91	19.4	23.0	Slurry pit, (depth: N/A†) Fully slatted	154.7 g day^{-1} AU^{-1}
Wi, *et al.* [5]	Fattening pig	0.79	29.0	31.7	Shallow pit (depth: 0.8 m) Fully slatted	162.0 g day^{-1} AU^{-1}
Ni, *et al.* [6]	Fattening pig	0.88	21.8	25.2	Deep pit (depth: 2.4 m), Fully slatted	145 g day^{-1} AU^{-1}
Heber, *et al.* [7]	Fattening pig	0.90–0.91	21.9	25.3	Deep pit (depth: 2.4 m), Fully slatted	146.7 g day^{-1} AU^{-1}
		0.92–0.93	20.0	24.1		121.8 g day^{-1} AU^{-1}
Philippe, *et al.* [8]	Fattening pig	0.76	19.2	20.2	Slurry pit (depth: N/A) Fully slatted	8.5 g day^{-1} $head^{-1}$
Van Ransbeeck, et al. [4]	Fattening pig	N/A	15.6	29.8	Slurry pit (depth: N/A) Fully slatted	6.0 g day^{-1} $head^{-1}$

† N/A = Not available.

Table S5. Seasonal ammonia emissions and manure accumulation in the slurry pit during different monitoring periods.

		Summer		Late autumn		Winter	
Day		0–6	7–14	0–7	8–15	0–7	8–15
Ammonia emission	g day^{-1} AU^{-1}	125.8 ± 7.6^a	96.2 ± 25.3^b	122.6 ± 42.2^a	127.4 ± 34.3^a	114.8 ± 20.5^a	99.4 ± 18.5^a
	g day^{-1} $head^{-1}$	19.4 ± 1.5^a	16.0 ± 3.9^b	18.7 ± 6.0^a	21.3 ± 5.5^a	15.8 ± 2.5^a	15.0 ± 2.7^a
Amount of manure accumulated in the slurry pit ton, (% of pit capacity)		16.2 ± 1.7, (7.2 ± 0.8)	21.8 ± 1.7, (9.7 ± 0.8)	14.8 ± 1.9, (6.6 ± 0.8)	20.9 ± 1.9, (9.3 ± 0.8)	8.7 ± 2.0, (3.9 ± 0.9)	15.4 ± 2.0, (6.8 ± 0.9)

^{a,b} Different superscripts in the same row meaning each group is significantly different ($p < 0.05$)

Table S6. Housing characteristics and manure handling practices in previous studies referenced in Table 2.

	Growth stage (Head)	Average body weight (kg)	Dimensions of each room (m^2)	Manure handling practice
Sun, et al. [3]	Fattening (240–257)	60–78	250	Slurry pit, (depth: N/A†) Fully slatted
Blunden, *et al.* [9]	Finisher (842−896)	38–88	744	Shallow pit, Pit recharge system (1 per week)
Costa and Guarino [10]	Gestation (37−42	N/A	85	Slurry pit, (depth: N/A) Fully slatted
	Fattening (343–345)		295	
Rumsey, *et al.* [11]	Fattening (476–995)	35–117	N/A	Shallow pit, (depth: 0.7 m), Flushing system (1 per week)

† N/A = Not available.

References

1. Choi, L.-y.; Lee, S.-y.; Jeong, H.; Park, J.; Hong, S.-w.; Kwon, K.-S.; Song, M. Ammonia and particulate matter emissions at a korean commercial pig farm and influencing factors. *Animals* **2023**, *13*, 3347.
2. Jerez, S.B.; Zhang, Y.; Wang, X. Spatial and temporal distributions of dust and ammonia concentrations in a swine building. *Transactions of the ASABE* **2011**, *54*, 1873-1891.
3. Sun, G.; Guo, H.; Peterson, J.; Predicala, B.; Laguë, C.J.J.o.t.A.; Association, W.M. Diurnal odor, ammonia, hydrogen sulfide, and carbon dioxide emission profiles of confined swine grower/finisher rooms. **2008**, *58*, 1434-1448.
4. Van Ransbeeck, N.; Van Langenhove, H.; Demeyer, P. Indoor concentrations and emissions factors of particulate matter, ammonia and greenhouse gases for pig fattening facilities. *Biosystems Engineering* **2013**, *116*, 518-528.
5. Wi, J.; Lee, S.; Kim, E.; Lee, M.; Koziel, J.A.; Ahn, H. Effects of treated manure conditions on ammonia and hydrogen sulfide emissions from a swine finishing barn equipped with semicontinuous pit recharge system in summer. *Atmosphere* **2020**, *11*, 713.
6. Ni, J.Q.; Heber, A.J.; Lim, T.T.; Diehl, C.A.; Duggirala, R.K.; Haymore, B.L.; Sutton, A.L. *Ammonia emission from a large mechanically-ventilated swine building during warm weather*; 0047-2425; Wiley Online Library: 2000.
7. Heber, A.; Ni, J.; Lim, T.; Diehl, C.; Sutton, A.; Duggirala, R.; Haymore, B.; Kelly, D.; Adamchuk, V.J.T.o.t.A. Effect of a manure additive on ammonia emission from swine finishing buildings. **2000**, *43*, 1895.
8. Philippe, F.-X.; Laitat, M.; Canart, B.; Vandenheede, M.; Nicks, B.J.L.S. Comparison of ammonia and greenhouse gas emissions during the fattening of pigs, kept either on fully slatted floor or on deep litter. **2007**, *111*, 144-152.
9. Blunden, J.; Aneja, V.P.; Westerman, P.W.J.A.e. Measurement and analysis of ammonia and hydrogen sulfide emissions from a mechanically ventilated swine confinement building in North Carolina. **2008**, *42*, 3315-3331.
10. Costa, A.; Guarino, M.J.A.E. Definition of yearly emission factor of dust and greenhouse gases

through continuous measurements in swine husbandry. **2009**, *43*, 1548-1556.
11. Rumsey, I.C.; Aneja, V.P.; Lonneman, W.A.J.A.e. Characterizing reduced sulfur compounds emissions from a swine concentrated animal feeding operation. **2014**, *94*, 458-466.
